# Supplementary material for: Mechanisms governing the pioneering and redistribution capabilities of the non-classical pioneer PU.1
Source: Nat Commun. 2020 Jan 21;11:402. doi: 10.1038/s41467-019-13960-2 (PMC6972792; doi:10.1038/s41467-019-13960-2)
Supplement: Supplementary file 7 — Source data [file 41467_2019_13960_MOESM7_ESM.zip › Source_Data/Figure6/Figure6B_MotifScanOutput/FLI1_PU.1/homerResults.html]

/misc/data/analysis/project\_PU1/CTV1/motifs/basic/peaksFLI1\_DSG\_PU1\_merged/final - Homer de novo Motif Results


# Homer *de novo* Motif Results (/misc/data/analysis/project\_PU1/CTV1/motifs/basic/peaksFLI1\_DSG\_PU1\_merged/final)

Known Motif Enrichment Results  
Gene Ontology Enrichment Results  
If Homer is having trouble matching a motif to a known motif, try copy/pasting the matrix file into
STAMP  
More information on motif finding results: HOMER
| Description of Results
| Tips
  
Total target sequences = 9949  
Total background sequences = 36485  
\* - possible false positive  

|  |  |  |  |  |  |  |  |  |
| --- | --- | --- | --- | --- | --- | --- | --- | --- |
| Rank | Motif | P-value | log P-pvalue | % of Targets | % of Background | STD(Bg STD) | Best Match/Details | Motif File |
| 1 | C T G A A G T C G C A T A C G T A G T C A G T C A C G T A T C G G C A T A G C T | 1e-2892 | -6.661e+03 | 82.24% | 19.96% | 43.8bp (74.7bp) | Etv2(ETS)/ES-ER71-ChIP-Seq(GSE59402)/Homer(0.967)(0.972) More Information \| Similar Motifs Found | motif file (matrix) |
| 2 | A G T C C G T A A G T C C G A T A C G T A G T C A G C T | 1e-639 | -1.473e+03 | 41.12% | 14.89% | 56.0bp (70.6bp) | PU.1(ETS)/ThioMac-PU.1-ChIP-Seq(GSE21512)/Homer(0.750) More Information \| Similar Motifs Found | motif file (matrix) |
| 3 | C G T A A T C G G C A T C T G A T C G A A G T C A G T C C T G A A G T C G C T A | 1e-592 | -1.365e+03 | 36.58% | 12.54% | 49.6bp (63.5bp) | RUNX2(Runt)/PCa-RUNX2-ChIP-Seq(GSE33889)/Homer(0.970) More Information \| Similar Motifs Found | motif file (matrix) |
| 4 | C T A G T C G A A T C G A C T G C G T A C G T A T A G C A G C T C G T A | 1e-396 | -9.124e+02 | 37.37% | 16.72% | 53.4bp (89.6bp) | SPIB/MA0081.1/Jaspar(0.724) More Information \| Similar Motifs Found | motif file (matrix) |
| 5 | G C A T A C T G T G C A A T C G C T A G G C T A G C T A C G T A A G T C T C A G A T C G | 1e-330 | -7.621e+02 | 17.89% | 4.99% | 53.8bp (66.7bp) | EWS:ERG-fusion(ETS)/CADO\_ES1-EWS:ERG-ChIP-Seq(SRA014231)/Homer(0.766) More Information \| Similar Motifs Found | motif file (matrix) |
| 6 | T C A G T A C G C T A G T C G A T C G A T C A G T C A G T G C A C T G A | 1e-273 | -6.295e+02 | 13.55% | 3.41% | 56.6bp (86.6bp) | RBM5(Znf)/Homo\_sapiens-RNCMPT00055-PBM/HughesRNA(0.897) More Information \| Similar Motifs Found | motif file (matrix) |
| 7 | A G T C G A C T A G C T A G T C G A C T A G C T A G T C G A C T A G C T A G T C G A C T A G C T A G T C | 1e-207 | -4.776e+02 | 50.25% | 33.24% | 50.1bp (85.7bp) | Unknown4/Arabidopsis-Promoters/Homer(0.765) More Information \| Similar Motifs Found | motif file (matrix) |
| 8 | T C G A T A G C G T C A C T A G A C T G C G T A A G C T C T A G G A C T A C T G A C T G T A C G | 1e-201 | -4.635e+02 | 7.75% | 1.42% | 46.0bp (57.6bp) | ETS:RUNX(ETS,Runt)/Jurkat-RUNX1-ChIP-Seq(GSE17954)/Homer(0.867) More Information \| Similar Motifs Found | motif file (matrix) |
| 9 | A C G T A C G T A G T C C G A T C A G T G T A C A T G C G C A T G T A C A C G T A T C G A G T C | 1e-169 | -3.903e+02 | 9.14% | 2.44% | 53.5bp (54.1bp) | SPIB/MA0081.1/Jaspar(0.755) More Information \| Similar Motifs Found | motif file (matrix) |
| 10 | C T G A T C G A C G T A C G T A C G T A C T A G A C T G A C T G C T A G C G T A C G T A T C G A G A C T C G A T | 1e-135 | -3.123e+02 | 6.33% | 1.45% | 51.1bp (70.3bp) | NDD1(MacIsaac)/Yeast(0.873) More Information \| Similar Motifs Found | motif file (matrix) |
| 11 | A C G T C G A T T A G C A G T C A C T G A G T C T A G C A T C G | 1e-123 | -2.832e+02 | 13.58% | 6.01% | 53.6bp (75.0bp) | HAL9/MA0311.1/Jaspar(0.777) More Information \| Similar Motifs Found | motif file (matrix) |
| 12 | T A G C G T A C G C T A G T A C C G T A C A T G T A C G T G C A C G T A G C T A T G A C | 1e-121 | -2.803e+02 | 4.32% | 0.70% | 52.3bp (74.0bp) | RAV1(RAV)/colamp-RAV1-DAP-Seq(GSE60143)/Homer(0.622) More Information \| Similar Motifs Found | motif file (matrix) |
| 13 | T C G A G A T C G T C A C T A G T A G C T G C A C G T A C T G A A T G C C G A T A C T G | 1e-118 | -2.730e+02 | 9.32% | 3.34% | 57.8bp (62.1bp) | prd/dmmpmm(Down)/fly(0.682) More Information \| Similar Motifs Found | motif file (matrix) |
| 14 | T C G A C T A G T C G A C T G A C T A G C T G A C G T A C T A G C T A G C G T A | 1e-115 | -2.653e+02 | 22.79% | 13.12% | 51.9bp (89.3bp) | SF2(RRM)/Drosophila\_melanogaster-RNCMPT00066-PBM/HughesRNA(0.758) More Information \| Similar Motifs Found | motif file (matrix) |
| 15 | A G T C C A T G T C G A A C G T A C T G C G T A T A G C A C G T A T G C C G T A A G T C | 1e-105 | -2.420e+02 | 7.32% | 2.40% | 53.2bp (64.2bp) | JunB(bZIP)/DendriticCells-Junb-ChIP-Seq(GSE36099)/Homer(0.979) More Information \| Similar Motifs Found | motif file (matrix) |
| 16 | G C A T C T G A C T G A G T A C G T C A C T A G G A T C | 1e-104 | -2.403e+02 | 57.16% | 44.88% | 57.6bp (67.0bp) | MYB65(MYB)/colamp-MYB65-DAP-Seq(GSE60143)/Homer(0.813) More Information \| Similar Motifs Found | motif file (matrix) |
| 17 | G T A C G A T C C A T G T G A C A G T C A G T C G T C A G T A C C G T A | 1e-103 | -2.378e+02 | 27.36% | 17.36% | 55.0bp (65.2bp) | Egr2(Zf)/Thymocytes-Egr2-ChIP-Seq(GSE34254)/Homer(0.881) More Information \| Similar Motifs Found | motif file (matrix) |
| 18 | C T G A G T A C C G T A C T A G T C A G G T C A C G T A C A T G T A G C G T C A C A G T T C A G | 1e-98 | -2.259e+02 | 4.37% | 0.95% | 57.5bp (67.6bp) | Ets1-distal(ETS)/CD4+-PolII-ChIP-Seq(Barski\_et\_al.)/Homer(0.754) More Information \| Similar Motifs Found | motif file (matrix) |
| 19 | T A G C G C T A A C T G C G T A A C G T C G T A T C G A T A C G T C G A | 1e-96 | -2.231e+02 | 15.15% | 7.86% | 53.9bp (65.9bp) | GATA5/MA0766.1/Jaspar(0.962) More Information \| Similar Motifs Found | motif file (matrix) |
| 20 | T C G A A T G C C G A T A C G T A G T C A C G T C T A G G A T C G C A T C G A T G A C T | 1e-83 | -1.926e+02 | 8.74% | 3.71% | 54.4bp (66.6bp) | SCL(bHLH)/HPC7-Scl-ChIP-Seq(GSE13511)/Homer(0.793) More Information \| Similar Motifs Found | motif file (matrix) |
| 21 | T G A C A G T C T G C A A T G C C G A T A C G T A G T C G T A C A C G T G T A C C G A T A C T G | 1e-78 | -1.803e+02 | 3.42% | 0.72% | 52.0bp (69.1bp) | PU.1(ETS)/ThioMac-PU.1-ChIP-Seq(GSE21512)/Homer(0.850) More Information \| Similar Motifs Found | motif file (matrix) |
| 22 | G A T C A C T G G T A C A C T G A C T G C G T A C G T A A C T G C G T A A C T G A C T G A T G C A C T G | 1e-55 | -1.282e+02 | 2.89% | 0.74% | 56.1bp (76.5bp) | Gabpa/MA0062.2/Jaspar(0.779) More Information \| Similar Motifs Found | motif file (matrix) |
| 23 | A G C T A G T C C G A T A C G T A G T C A T G C C G A T A T G C A T G C A G C T T G A C A G T C C G A T | 1e-55 | -1.280e+02 | 3.88% | 1.26% | 53.0bp (57.8bp) | SRSF1(RRM)/Homo\_sapiens-RNCMPT00163-PBM/HughesRNA(0.697) More Information \| Similar Motifs Found | motif file (matrix) |
| 24 | C A T G A C T G A T C G A T C G A T C G T C A G A C T G | 1e-53 | -1.221e+02 | 24.48% | 17.49% | 56.2bp (69.9bp) | ZNF740/MA0753.1/Jaspar(0.916) More Information \| Similar Motifs Found | motif file (matrix) |
| 25 | A C T G A C T G T C G A A C T G A C T G T C G A C T G A T C A G T C G A G C T A C G T A C T G A | 1e-52 | -1.209e+02 | 5.15% | 2.09% | 57.5bp (74.3bp) | RLR1/RLR1\_YPD/[](Harbison)/Yeast(0.736) More Information \| Similar Motifs Found | motif file (matrix) |
| 26 | A G C T A C G T G A C T C G A T C A G T C G T A A C G T T A G C C G A T | 1e-52 | -1.200e+02 | 9.64% | 5.26% | 54.0bp (67.4bp) | GATA3/MA0037.2/Jaspar(0.860) More Information \| Similar Motifs Found | motif file (matrix) |
| 27 | C T A G A C T G A C T G A G T C A C T G A C T G C T A G C T A G A G T C | 1e-51 | -1.177e+02 | 7.42% | 3.67% | 56.6bp (67.1bp) | Sp1(Zf)/Promoter/Homer(0.895) More Information \| Similar Motifs Found | motif file (matrix) |
| 28 | A C T G A G T C C G T A C T A G A T C G G T C A C G T A C T A G C T A G T C G A | 1e-47 | -1.098e+02 | 4.17% | 1.59% | 51.6bp (72.5bp) | SOK2/SOK2\_BUT14/4-SUT1(Harbison)/Yeast(0.785) More Information \| Similar Motifs Found | motif file (matrix) |
| 29 | A C G T T A C G A G C T C G A T A T C G G A T C A C G T A G T C A G C T C G A T G T A C A C G T | 1e-44 | -1.020e+02 | 3.04% | 0.98% | 59.9bp (82.5bp) | PABPN1(RRM)/Homo\_sapiens-RNCMPT00157-PBM/HughesRNA(0.716) More Information \| Similar Motifs Found | motif file (matrix) |
| 30 | T G C A C G T A T A C G G T C A T G C A T C A G T G C A C T G A T A C G G C A T A C T G T C A G C G A T A C T G | 1e-42 | -9.809e+01 | 2.77% | 0.86% | 52.1bp (63.4bp) | SpiB(ETS)/OCILY3-SPIB-ChIP-Seq(GSE56857)/Homer(0.720) More Information \| Similar Motifs Found | motif file (matrix) |
| 31 | C G A T A C G T A G T C C G A T A G T C G T A C G C A T A T G C G A C T A C G T A G T C A G T C A G C T | 1e-42 | -9.704e+01 | 4.18% | 1.71% | 59.6bp (83.6bp) | LIN28A(CSD,Znf)/Homo\_sapiens-RNCMPT00036-PBM/HughesRNA(0.700) More Information \| Similar Motifs Found | motif file (matrix) |
| 32 | A G C T A C G T A G T C A C G T C A T G A G C T A C G T A C G T G T A C G A C T T A G C G A C T A G C T A C G T | 1e-37 | -8.606e+01 | 3.16% | 1.18% | 53.9bp (63.0bp) | br-Z4/dmmpmm(Bergman)/fly(0.648) More Information \| Similar Motifs Found | motif file (matrix) |
| 33 | C G A T C T A G T C A G G A T C T G C A C T A G A G C T G A C T | 1e-31 | -7.231e+01 | 13.64% | 9.47% | 58.0bp (65.4bp) | Myb/MA0100.2/Jaspar(0.938) More Information \| Similar Motifs Found | motif file (matrix) |
| 34 | T C A G T G A C T C A G A G T C G T C A A G C T A C T G A G T C A C T G G A T C C G T A | 1e-29 | -6.768e+01 | 2.76% | 1.10% | 56.4bp (65.6bp) | NRF(NRF)/Promoter/Homer(0.966) More Information \| Similar Motifs Found | motif file (matrix) |
| 35 | C G A T A G T C T A G C G A T C T A G C G T A C G A C T G A C T A G T C G C A T C G A T G A T C | 1e-28 | -6.550e+01 | 1.49% | 0.38% | 61.2bp (56.2bp) | ZML2(C2C2gata)/col-ZML2-DAP-Seq(GSE60143)/Homer(0.700) More Information \| Similar Motifs Found | motif file (matrix) |
| 36 | A T C G C G T A A G T C A C T G A C G T A T G C C G T A A G T C | 1e-27 | -6.269e+01 | 3.62% | 1.72% | 57.3bp (60.9bp) | HY5(bZIP)/colamp-HY5-DAP-Seq(GSE60143)/Homer(0.900) More Information \| Similar Motifs Found | motif file (matrix) |
| 37 | T A G C A T C G A T G C G C A T C G T A C A T G G A T C C A T G T G A C A T C G | 1e-24 | -5.563e+01 | 2.88% | 1.31% | 58.5bp (66.2bp) | STP3/MA0396.1/Jaspar(0.864) More Information \| Similar Motifs Found | motif file (matrix) |
| 38 | G T C A C T G A C A T G A C T G T C G A C T A G G C T A G C T A C T A G A C T G G C T A C T A G T C G A | 1e-23 | -5.368e+01 | 1.43% | 0.43% | 54.8bp (53.6bp) | eor-1/MA0543.1/Jaspar(0.687) More Information \| Similar Motifs Found | motif file (matrix) |
| 39 | C G A T A G T C G T A C A C G T G T A C A T G C A C G T A C G T A G T C A C G T A G T C A G T C | 1e-23 | -5.314e+01 | 1.71% | 0.59% | 54.2bp (65.8bp) | REF2(RRM)/Drosophila\_melanogaster-RNCMPT00059-PBM/HughesRNA(0.733) More Information \| Similar Motifs Found | motif file (matrix) |
| 40 | T G C A T G A C G T A C T A C G A C T G C T G A C T G A G T C A A G T C A G T C A C G T | 1e-21 | -5.028e+01 | 1.05% | 0.25% | 47.8bp (74.2bp) | YRM1/MA0438.1/Jaspar(0.722) More Information \| Similar Motifs Found | motif file (matrix) |
| 41 | C G T A T C G A A C T G C T G A A C T G G T C A C G T A G T C A C T A G C A T G C T A G A T C G G T C A C G T A | 1e-20 | -4.776e+01 | 0.95% | 0.22% | 49.9bp (62.2bp) | IRF1/MA0050.2/Jaspar(0.655) More Information \| Similar Motifs Found | motif file (matrix) |
| 42 | A C G T A T G C C G A T A C G T A G T C G A C T T A C G A T G C C G A T A C G T A C G T A G T C | 1e-20 | -4.700e+01 | 1.50% | 0.51% | 58.8bp (71.8bp) | RLR1/RLR1\_YPD/[](Harbison)/Yeast(0.676) More Information \| Similar Motifs Found | motif file (matrix) |
| 43 | C T G A T C G A T C A G A C T G A C T G C G T A G T C A A C T G A G T C G A C T G C A T | 1e-19 | -4.421e+01 | 2.12% | 0.93% | 52.9bp (52.5bp) | GCR2(MacIsaac)/Yeast(0.835) More Information \| Similar Motifs Found | motif file (matrix) |
| 44 | G A C T T G A C A G C T A G T C A C T G A G T C A C T G G T C A A C T G T C G A G T C A A T G C | 1e-18 | -4.374e+01 | 0.82% | 0.18% | 58.6bp (70.9bp) | GFX(?)/Promoter/Homer(0.976) More Information \| Similar Motifs Found | motif file (matrix) |
| 45 | A G C T A T C G C G A T A C G T A C T G A G T C C G T A G T C A | 1e-18 | -4.251e+01 | 5.10% | 3.15% | 57.8bp (65.5bp) | CEBP:AP1(bZIP)/ThioMac-CEBPb-ChIP-Seq(GSE21512)/Homer(0.927) More Information \| Similar Motifs Found | motif file (matrix) |
| 46 | C G T A G T C A T C G A G T C A C G A T A C T G A T C G G T A C C T A G C T A G A G T C | 1e-18 | -4.243e+01 | 1.18% | 0.37% | 53.4bp (66.5bp) | YY1(Zf)/Promoter/Homer(0.941) More Information \| Similar Motifs Found | motif file (matrix) |
| 47 | C T A G C G T A C T A G A T C G A G T C A G T C A C G T A G T C G C T A C T A G T A C G C T G A C T G A C T G A | 1e-15 | -3.609e+01 | 0.89% | 0.26% | 50.6bp (57.7bp) | ZFX(Zf)/mES-Zfx-ChIP-Seq(GSE11431)/Homer(0.716) More Information \| Similar Motifs Found | motif file (matrix) |
| 48 | A C G T A G C T T A G C G C A T A C G T A T C G A C G T A G C T A C G T A C G T A G T C A G T C C G A T | 1e-14 | -3.353e+01 | 0.76% | 0.21% | 53.4bp (58.2bp) | PB0141.1\_Isgf3g\_2/Jaspar(0.656) More Information \| Similar Motifs Found | motif file (matrix) |
| 49 | A C T G A C T G C T A G C T A G C T G A T C G A C T G A A C G T C T A G C T G A C T A G C G T A | 1e-14 | -3.321e+01 | 1.42% | 0.59% | 54.6bp (70.7bp) | Gata4/MA0482.1/Jaspar(0.626) More Information \| Similar Motifs Found | motif file (matrix) |
| 50 | T C G A C T A G A C G T A G T C G T C A A C T G A C G T C G A T T A G C C A G T | 1e-13 | -2.996e+01 | 1.38% | 0.60% | 64.7bp (62.8bp) | MYB.PH3(2)(MYB)/Petunia hybrida/AthaMap(0.678) More Information \| Similar Motifs Found | motif file (matrix) |
| 51 | C A G T T C G A C G T A T C A G G A C T A C G T C A G T A T G C G T A C G C A T T C A G C G T A C T A G T C A G | 1e-12 | -2.951e+01 | 0.60% | 0.15% | 52.7bp (54.4bp) | EXC-7(RRM)/Caenorhabditis\_elegans-RNCMPT00014-PBM/HughesRNA(0.587) More Information \| Similar Motifs Found | motif file (matrix) |
| 52 | G A T C G A C T A G T C C T A G C A G T T A C G C T G A T C A G T G C A C T G A G A T C | 1e-12 | -2.946e+01 | 0.90% | 0.31% | 62.9bp (64.9bp) | Su(H)/dmmpmm(Papatsenko)/fly(0.796) More Information \| Similar Motifs Found | motif file (matrix) |
| 53 | A T G C C G T A C G A T G C A T A C G T A T C G T G A C A C T G A C T G C G T A | 1e-12 | -2.912e+01 | 1.24% | 0.51% | 58.3bp (73.6bp) | YER184C/MA0424.1/Jaspar(0.696) More Information \| Similar Motifs Found | motif file (matrix) |
| 54 | A G T C A C G T A G T C A G T C A C G T A G T C A G T C | 1e-12 | -2.904e+01 | 14.81% | 12.04% | 55.9bp (77.3bp) | SRSF1(RRM)/Homo\_sapiens-RNCMPT00163-PBM/HughesRNA(0.958) More Information \| Similar Motifs Found | motif file (matrix) |
| 55 | A C T G A T G C C G T A G T C A G T C A A G T C C T G A A C T G G T C A | 1e-12 | -2.860e+01 | 4.38% | 2.90% | 58.1bp (64.1bp) | Foxo1(Forkhead)/RAW-Foxo1-ChIP-Seq(Fan\_et\_al.)/Homer(0.714) More Information \| Similar Motifs Found | motif file (matrix) |
| 56 | A G T C T A C G A T G C A T G C A G C T A G C T A G T C A G C T A C G T A G T C A G T C A T C G A C T G T A G C | 1e-12 | -2.785e+01 | 0.97% | 0.36% | 51.5bp (61.6bp) | Eip74EF/MA0026.1/Jaspar(0.795) More Information \| Similar Motifs Found | motif file (matrix) |
